# Overexpression of a novel candidate oncogene KIF14 correlates with tumor progression and poor prognosis in prostate cancer

**DOI:** 10.18632/oncotarget.17564

**Published:** 2017-05-02

**Authors:** Yixiang Zhang, Yeqing Yuan, Pei Liang, Zhaoxia Zhang, Xiaojing Guo, Ligang Xia, Yingying Zhao, Xing-Sheng Shu, Shengkun Sun, Ying Ying, Yingduan Cheng

**Affiliations:** ^1^ Department of Urology, The Second Affiliated Hospital of Jinan University, Shenzhen People's Hospital, Shenzhen, Guangdong, People's Republic of China; ^2^ Department of Urology, David Geffen School of Medicine, University of California Los Angeles, Los Angeles, California, USA; ^3^ Department of Pediatrics, The Second Affiliated Hospital of Jinan University, Shenzhen People's Hospital, Shenzhen, Guangdong, People's Republic of China; ^4^ Department of Pathology, The Second Affiliated Hospital of Jinan University, Shenzhen People's Hospital, Shenzhen, Guangdong, People's Republic of China; ^5^ Department of Gastrointestinal Surgery, The Second Affiliated Hospital of Jinan University, Shenzhen People's Hospital, Shenzhen, Guangdong, People's Republic of China; ^6^ Department of Physiology, School of Basic Medical Sciences, Shenzhen University Health Sciences Center, Shenzhen, Guangdong, People's Republic of China; ^7^ Institute of Molecular Medicine, Health Science Center, Shenzhen University, Shenzhen, Guangdong, People's Republic of China; ^8^ Department of Urology, Chinese PLA General Hospital, Beijing, People's Republic of China

**Keywords:** KIF14, prostate cancer, apoptosis, proliferation, G2 arrest

## Abstract

Prostate cancer (PCa) is the second leading cause of death from cancer in men. The mechanism underlying tumorigenesis and development of PCa is largely unknown. Here, we identified Kinesin family member 14 (KIF14) as a novel candidate oncogene in PCa. We found that KIF14 was overexpressed in multiple PCa cell lines and primary PCa tissues. Knockdown of *KIF14* in DU145 and PC3 prostate cancer cells suppressed cell proliferation, induced cell cycle arrest and apoptosis. Transcriptome analysis by RNA-sequencing demonstrated that *KIF4* suppression led to transcriptional changes of genes involved in p53 and TGF-beta signaling pathway. In addition, upregulated expression of *GADD45A, GADD45B, p21, PIDD* and *Shisa5*, which contribute to growth arrest and apoptosis induction, and downregulated *CCNB1* that promotes cell cycle progression were confirmed by quantitative real-time PCR after *KIF4* knockdown. We further found that KIF14 protein level was positively correlated with T stage and Gleason Score. Patients with higher KIF14 expression had shorter overall survival time than those with lower KIF14 expression. Thus, our data indicate that KIF14 could act as a potential oncogene that contributes to tumor progression and poor prognosis in PCa, which may represent a novel and useful prognostic biomarker for PCa.

## INTRODUCTION

Prostate cancer (PCa) is one of the most commonly diagnosed malignant tumor and the second leading cause of cancer death among men in the United States. In 2016, about 180,890 new PCa cases were diagnosed and 26,120 PCa deaths were occurred in USA [1]. About one-third of patients with organ-confined PCa fail surgical treatment and progress to aggressive or metastatic lethal disease in 10 years [2]. Thus, understanding mechanisms underlying the tumorigenesis and development of PCa is urgently needed for early diagnosis, risk stratification and selection of therapy strategies against PCa. However, despite the impressive progress in discovery of genomic alterations in PCa, novel genomic bio-markers and proteins still remain to be identified.

The kinesin family proteins (KIFs) are ATP- and microtubule-associated motor proteins involved in cell division, microtubule polymer dynamics, intracellular transportation and signal transduction [3]. Kinesin family member 14 (KIF14) contains a C-terminal citron kinase binding region and an N-terminal protein regulating cytokinesis binding region [4, 5], and was found to bind to chromatin and microtubules during the formation of the bipolar spindle [4, 6]. Elevated *KIF14* expression can induce rapid and error-prone mitosis, leading to aneuploidy during tumorigenesis [6]. Although the exact molecular functions of KIF14 are still under investigation, increasing evidences has implicated *KIF14* as an oncogene whose overexpression has been found in multiple types of tumors, including hepatocellular carcinoma, lung cancer, breast cancer, glioma, laryngeal carcinoma and ovarian cancer, etc [6–12]. Moreover, *KIF14* levels are prognostic for the outcome of hepatocellular carcinoma and ovarian cancer, where *KIF14* overexpression enhances tumor growth, while its knockdown decreases tumorigenicity *in vitro* and in xenografts [13]. However, the expression of *KIF14* and its role in prostate carcinoma has not been reported thus far.

In this study, we found that *KIF14* was upregulated in human PCa cell lines and primary PCa tissues. Knockdown of *KIF14* in DU145 and PC3 prostate cancer cells reduced cell proliferation, induced cell cycle arrest and apoptosis. Transcriptome analysis by RNA-sequencing demonstrated that *KIF4* suppression led to transcriptional changes of genes involved in p53 and TGF-beta signaling pathway. In addition, gene expression of a few of cell cycle regulated proteins (GADD45A, GADD45B, p21 and CCNB1) and apoptotic proteins (PIDD and SHISA5) were confirmed by quantitative real-time PCR. We further found that KIF14 expression was significantly upregulated in primary human PCa specimens. KIF14 protein level was positively correlated with clinical stage and Gleason Score. Patients with higher KIF14 expression had shorter 5-year overall survival time than those with lower KIF14 expression. Thus, our data indicate that KIF14 overexpression contributes to prostate tumor progression and is associated with poor prognosis for PCa patients, which could be served as a novel and useful prognostic indicator for PCa.

## RESULTS

### KIF14 was overexpressed in PCa

**Figure 1:**
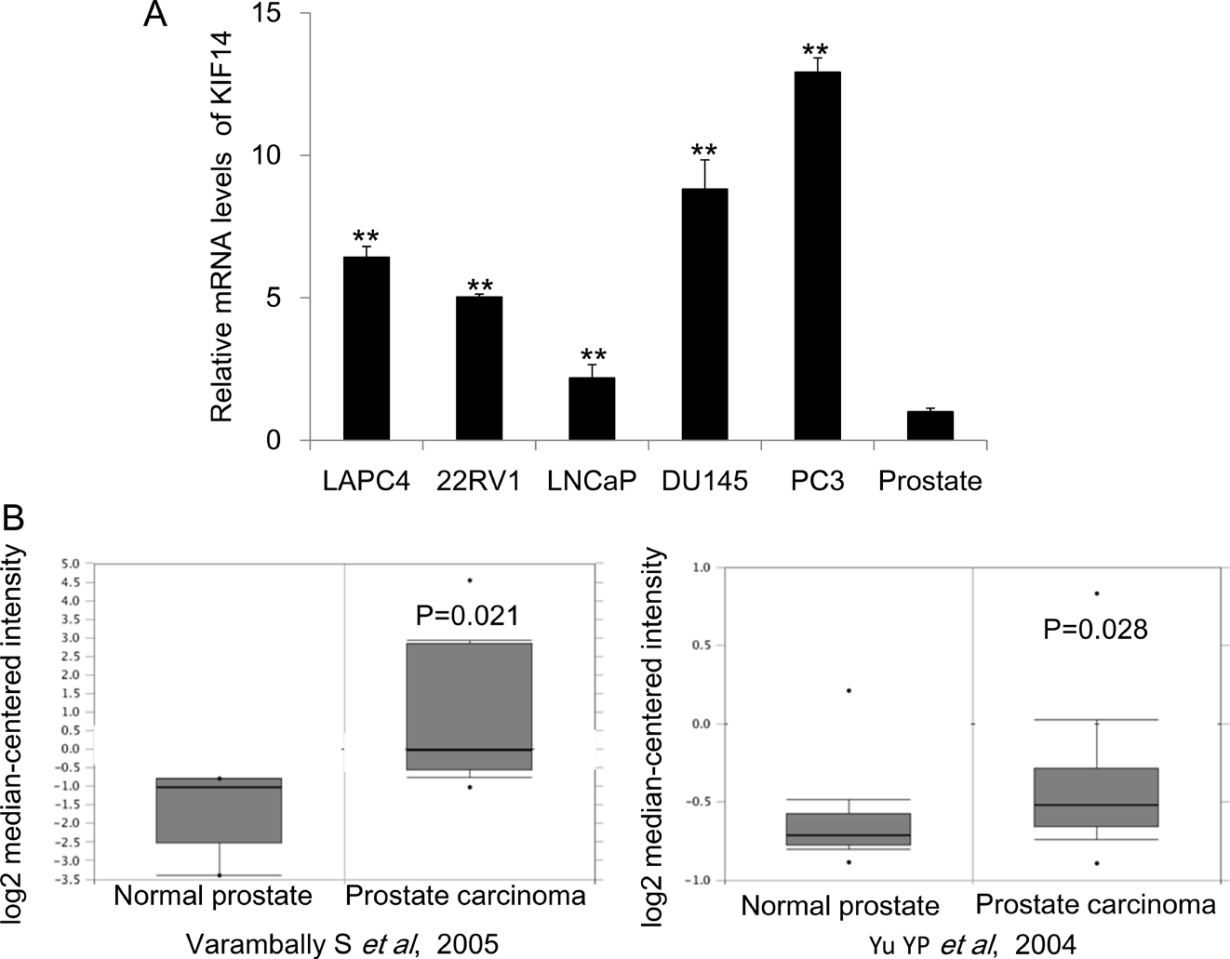
Expression levels of KIF14 in prostate cancer (**A**) Expression levels of KIF14 in a serial of prostate cancer cell lines and normal prostate tissue. *P < 0.05, ^*^P < 0.01. (**B**) Increased expression of KIF14 in studies by Varambally et al. (left) and Yu et al. (right) as obtained from the Oncomine cancer database.

Previous studies suggested that KIF14 was increased in multiple cancer including hepatocellular carcinoma (HCC), lung cancer, breast cancer, glioma, laryngeal carcinoma and ovarian cancer [6–12]. However, the role of KIF14 in prostate cancer remains unknown yet. We first checked the gene expression of *KIF14* in a serial PCa cell lines and normal prostate tissue and found that *KIF14* was significantly upregulated in PCa cells as compared to that in normal prostate tissue (Figure 1A). We further performed a summary for *KIF14* gene expression extracted from to the Oncomine database, a cancer microarray database and Integrated Data-Mining Platform [14]. A fold-change of 2 for *KIF14* gene expression compared to control was required for criterion. Based on analysis on two datasets derived from samples of patients with PCa [15, 16], we determined that *KIF14* is highly overexpressed in prostate carcinoma as compared with normal prostate tissues (Figure 1B). Thus, the overexpression of *KIF14* in multiple PCa cell lines and primary PCa suggested that *KIF14* might be a potential oncogene in PCa.

### Inhibition of KIF14 leads to G2 arrest and reduced proliferation in PCa cells

**Figure 2:**
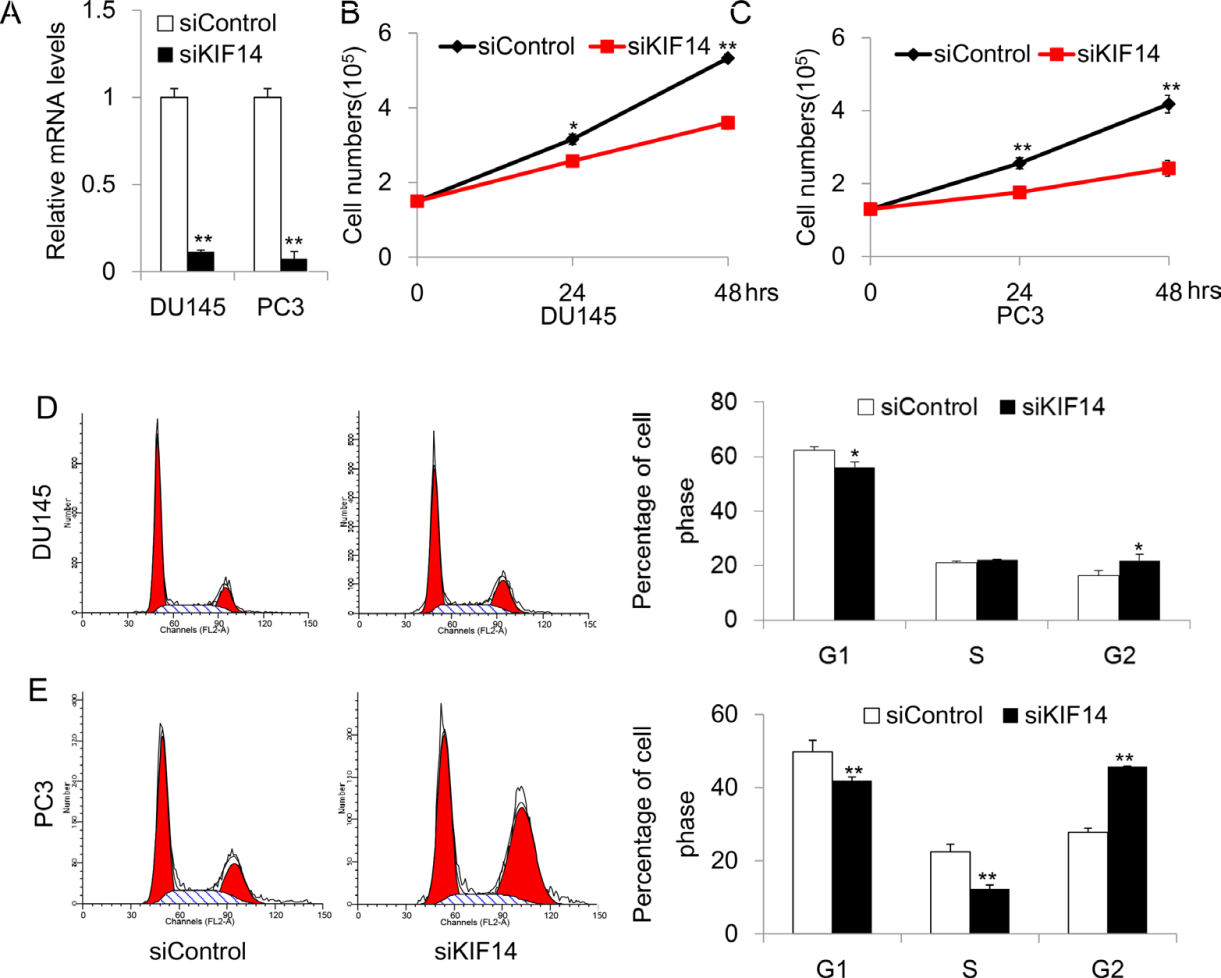
Knockdown of KIF14 inhibits cell proliferation and induce G2 arrest in DU145 and PC3 cell lines (**A**) Quantitative real-time PCR analysis of knockdown efficacy of *KIF14* in DU145 and PC3. *P < 0.05, ^*^P < 0.01.(**B**–**C**) Knockdown of *KIF14* resulted in G2 arrest both in DU145 and PC3 cell lines. *P < 0.05, ^*^P < 0.01. (**D**–**E**) Loss of *KIF14* inhibits cell growth in DU145 and PC3 cells. *P < 0.05, ^*^P < 0.01.

To explore the possible mechanism of *KIF14* as an oncogene, we adopted a small interfering RNA (siRNA) approach to knockdown *KIF14* and examined the effect of *KIF14* on cell cycle distribution and proliferation. In the experimental set-up, cell lines of DU145 and PC3, where *KIF14* was markedly upregulated as compared to normal control, were chosen for the functional study. The knockdown efficiency was first validated after 48 h transfection with siRNA. Quantitative real-time PCR analysis showed that the mRNA levels of *KIF14* were reduced by 89 ± 1% (*P* < 0.01) and 91 ± 5% (*P* < 0.01) in DU145 and PC3, respectively (Figure 2A). We examined whether reduced *KIF14* expression would affect prostate cancer cell growth by counting the cell numbers for three constitutive days after siRNA transfection. DU145 and PC3 cells exhibited reduced proliferation after KIF14 expression was knocked down (Figure 2B and Figure 2C). Flow cytometric analysis of propidium iodide (PI)-stained cells revealed that cells accumulated and arrested in G2 as evidenced by a significant increase in the fraction of cells in G2 in siKIF14-trasnfected DU145 and PC3 cells as compared to that in scramble control transfected cells (Figure 2D and Figure 2E), indicating that loss of *KIF14* induces G2 arrest in PCa.

### Knockdown of KIF14 leads to apoptosis of PCa cells

**Figure 3:**
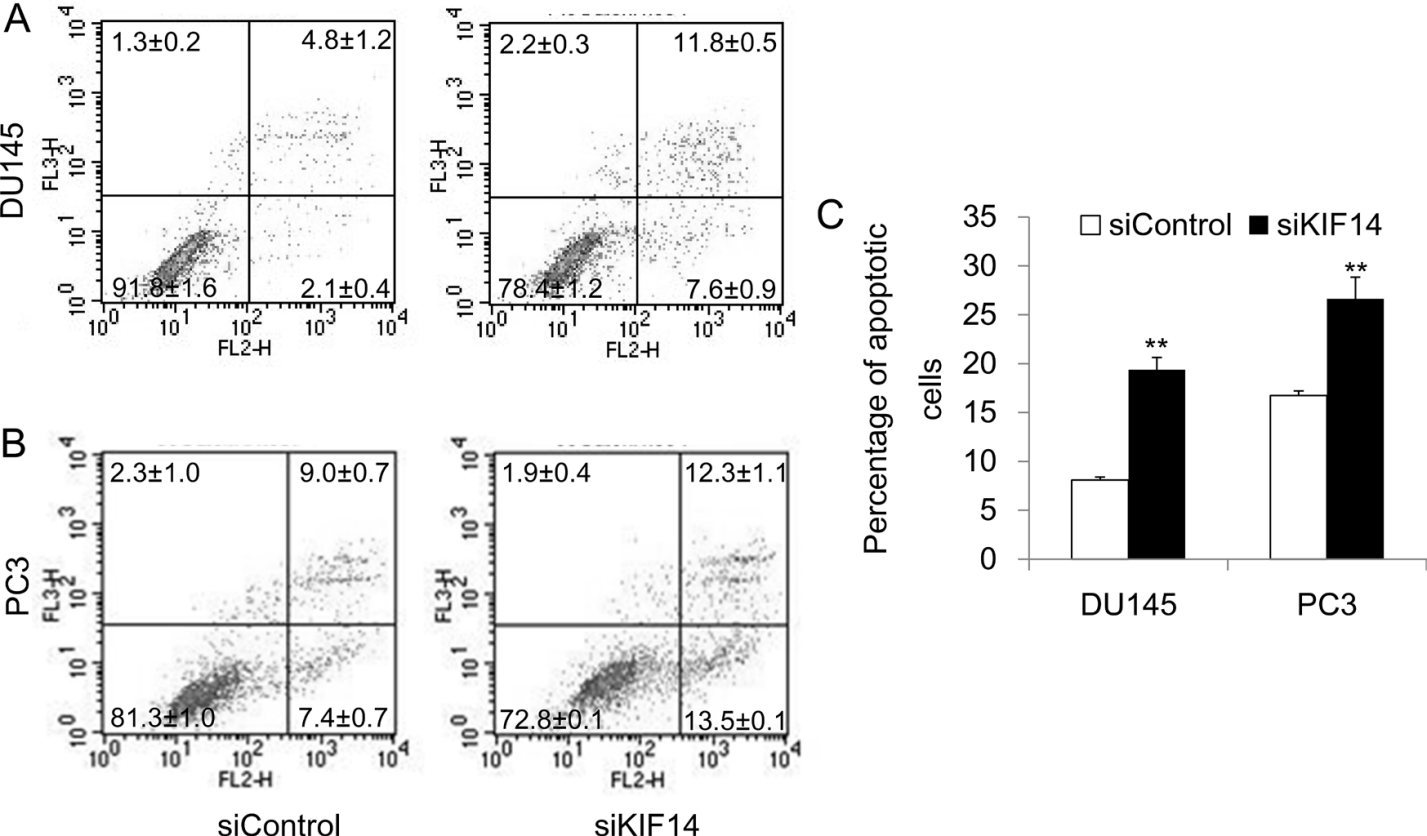
Knockdown of KIF14 induces apoptosis (**A**–**B**) Knockdown of *KIF14* resulted in apoptosis both in DU145 and PC3 cell lines as determined by flow cytometric analysis of Annexin V-PE/7-AAD-stained cells. (**C**) Statistical results of percentage of apoptotic cells in DU145 and PC3 cell lines. *P < 0.05, ^*^P < 0.01.

We further examined the effect of *KIF14* on apoptosis. Flow cytometric analysis of Annexin V-PE/7-AAD-stained cells after 48 hours after siRNA transfection discovered that loss of *KIF14* produced more apoptotic cells of DU145 and PC3 (Figure 3A–3C). Taken together, these data indicate that *KIF14* overexpression is necessary for G2/M cell cycle progression, promotion of cell proliferation, and prevention of cell apoptosis, which ultimately leads to indefinite growth of prostate cancer cells.

### KIF14 regulated genes involved in cell cycle and apoptosis

**Figure 4:**
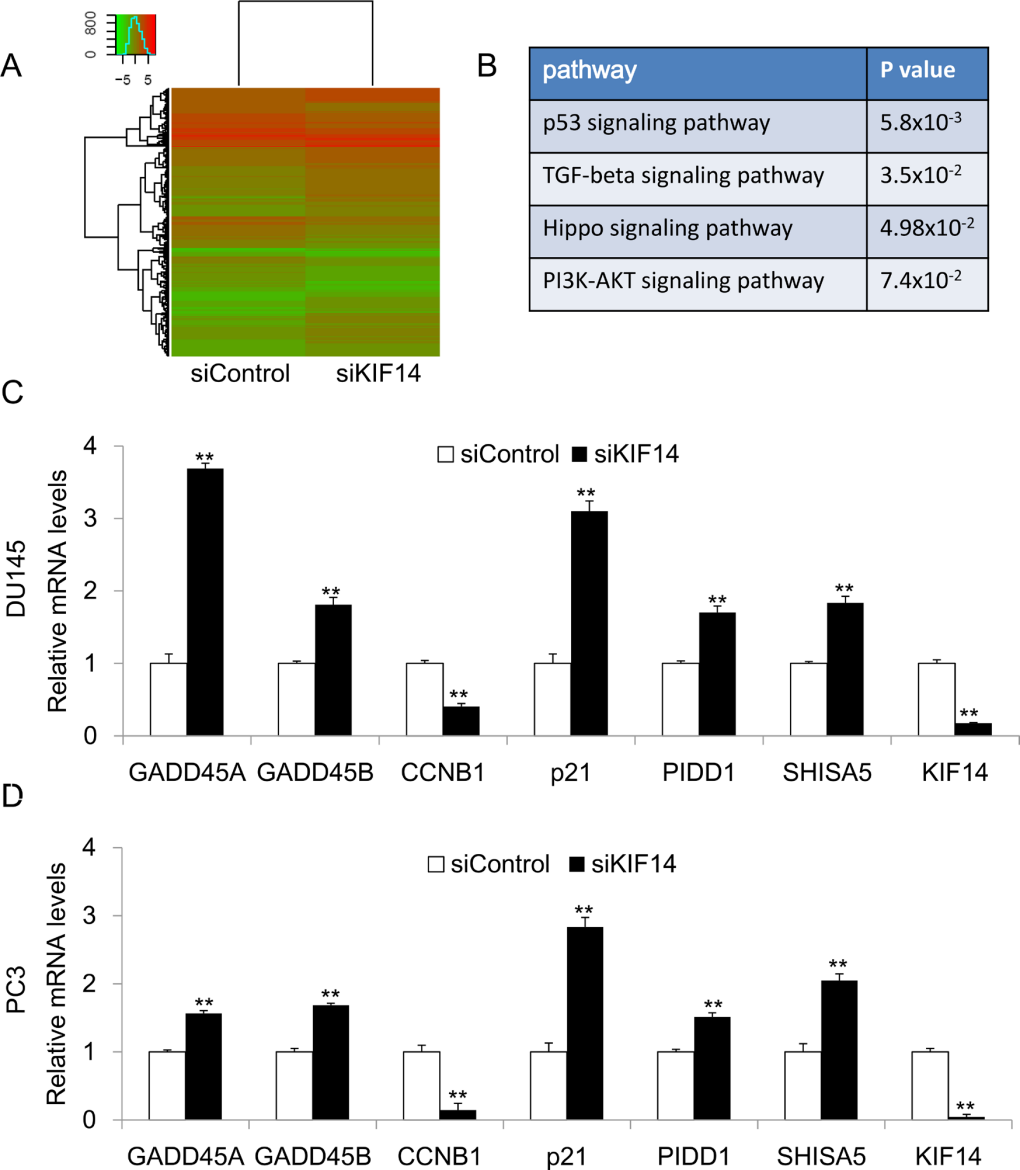
KIF14 downstream gene analysis (**A**) Heatmap of RNA-sequencing results from *KIF14* knockdown DU145 cells and control DU145 cells. (**B**) KEGG Pathway enrichment analysis for RNA sequencing analysis. (**C**–**D**) Confirmation of differentially expressed target genes in DU145 and PC3 cells by quantitative real-time PCR. *P < 0.05, ^*^P < 0.01.

Our functional study suggested that *KIF14* is a potential oncogene in PCa. To explore the underlying mechanism by which *KIF14* exerts oncogenic function in PCa, we performed RNA-Sequencing analysis to identify genes that were differentially expressed in *KIF14* knockdown DU145 cells and control DU145 cells. Genes with 2 fold changes were considered as significant (Figure 4A). Firstly, we analyzed the candidate genes by KEGG (Kyoto Encyclopedia of Genes and Genomes) Pathway enrichment analysis. We found that knockdown of *KIF14* led to transcriptional changes of genes involved in p53, TGF-beta, Hippo and PI3K-AKT signaling pathways (Figure 4B). The involvement of these cancer related pathway indicated that *KIF14* has functional role in carcinogenesis of PCa. We further confirmed the expression of potential *KIF14* target gene by quantitative real-time PCR. In both DU145 and PC3 cells, knockdown of *KIF14* upregulated the gene expression of *GADD45A*, *GADD45B*, *p21*, PIDD and *Shisa5*, which contribute to growth arrest and apoptosis induction, while downregulated *CCNB1* that promotes cell cycle progression (Figure 4C and Figure 4D). Our data suggested that *KIF14* may function as a candidate oncogene through regulating genes involved in cell cycle modulation and apoptosis, thus leading to tumor progression in PCa.

### KIF14 was overexpressed in primary PCa and positively correlated with clinical stage in patients with PCa

**Figure 5:**
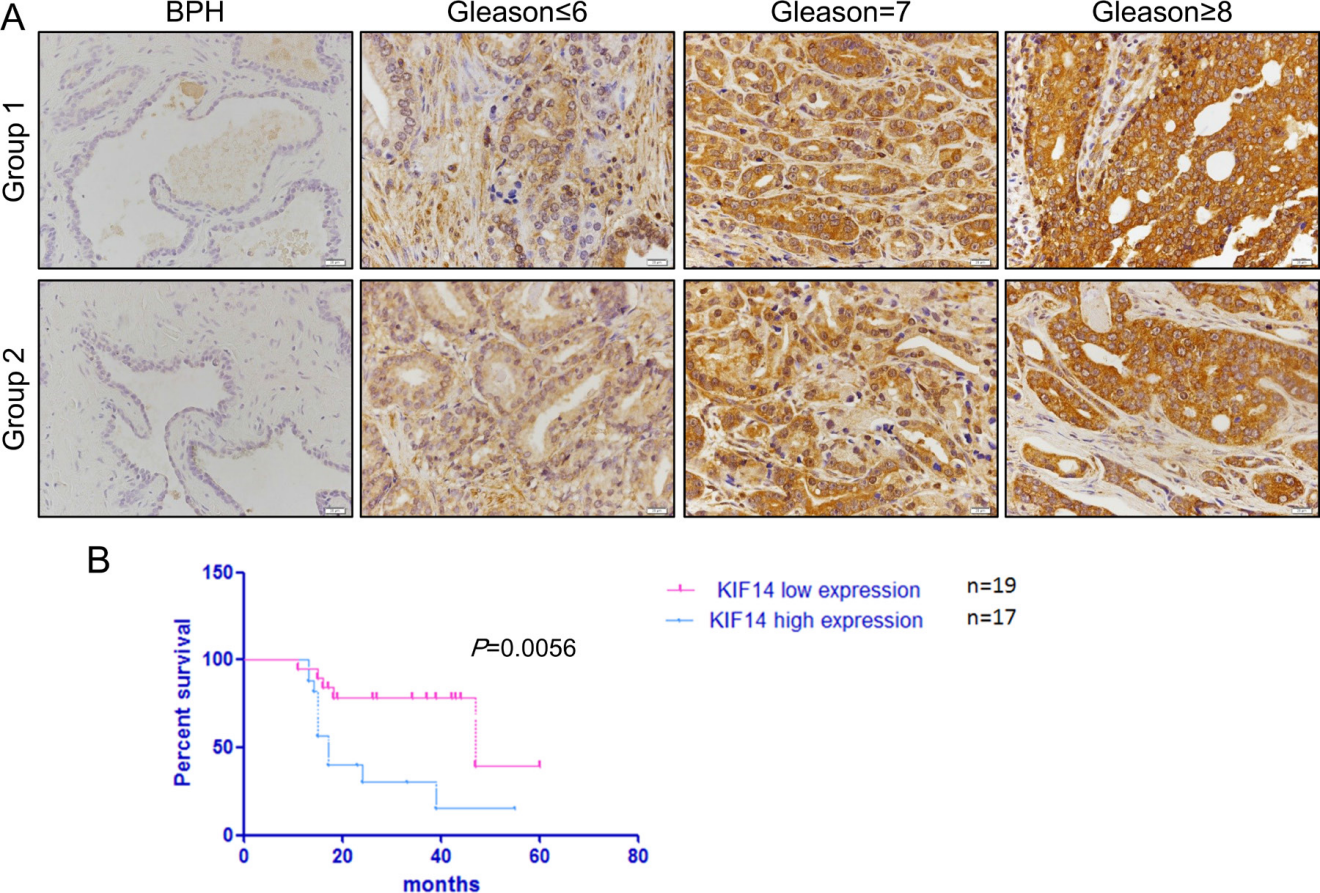
IHC analysis of KIF14 in prostate cancer patient samples (**A**) Representative IHC image of KIF14 in primary PCa samples. KIF14 was upregulated in patient samples and positively correlated with histological grade of primary PCa. (**B**) Kaplan-Meier survival curve for the correlation of KIF14 expression and 5-year overall survival of patients with PCa. Survival analysis was performed for KIF14 in a cohort of 19 low expression patients and 17 high expression patients, using a log-rank test *P* = 0.0056.

Our study suggested that *KIF14* was upregulated in PCa cell lines. We further investigated whether KIF14 was overexpressed in primary human PCa specimens by immunohistochemical staining. KIF14 was highly expressed in 57% (37/65) primary PCa tissues, while it was either absent or weakly expressed in another 20 BPH patients (20/25) (Table 1). Thus, KIF14 was significantly overexpressed in primary human PCa (*P* = 0.0017, Table 1). To further clarify the relationship between KIF14 expression and PCa progression, we investigated its expression and clinicopathological variables in 65 patients (Table 2). KIF14 expression was positively correlated with histological grade (Figure 5A). Moreover, KIF14 expression was significantly correlated with clinical stage (*P* = 0.0002) and Gleason score (*P* = 0.0041). However, no correlation was found between KIF14 expression and age and metastasis.

**Table 1:** Expression of KIF14 in BPH and PCa tissues

Type	Case Number	Low Expression	High Expression	*P*
BPH	25	20	5	0.0017
PCa	65	28	37	

In 20/25 BPH specimens, KIF14 was either absent or weakly expressed. However, the PCa tissue samples (37/65) were scored strong positive or moderate positive expression. The difference was significant. (*P* < 0.05).

**Table 2:** Relationship between clinicopathological variables and KIF14 expression level in PCa patients

Classification	Number	Low expression, *n*	High expression, *n*	*P*
Age (year)				0.7171
< 60	8	4	4	
≥ 60	57	24	33	
Primary tumor				0.0002
T1∼T2	31	22	12	
T3∼T4	34	6	25	
Metastasis				0.1232
−	47	23	24	
+	18	5	13	
Gleason Score				0.0041
≤ 6	14	9	5	
7	23	12	11	
≥ 8	28	7	21	

low expression including no(−) and weak(+) staining,

high expression including moderate (++) and strong (+++) staining.

Metastasis including lymph node and Distant metastasis.

### Prognostic significance of KIF4 expression

Clinical Gleason Score 8–10 is a negative independent prognostic factor on the progression free survival. In the present study, whereas 36% (5/14) of patients with Gleason Score≤6 showed low KIF14 expression, 75% (21/28) of men with Gleason Score of 8–10 displayed high KIF14 expression (*P* = 0.0041, Table 2), indicating a positive correlation of KIF14 overexpression with poor prognosis. Moreover, Kaplan-Meier analysis demonstrated that patients with higher KIF14 expression had a significantly shorter 5-year overall survival time (Figure 5B). Thus, these data emphasize the close association between KIF14 expression and prognosis in patients with PCa.

## DISCUSSION

Tumorigenesis is a complex process which contains lots of abnormal disruptions of tumor suppressor genes, oncogenes and their related signaling pathways [17–26]. PCa is one of the most common non-cutaneous malignancies among men worldwide with a peak incidence in men of ∼ 70 years [27]. Though some of the prostate cancer patients are no need to exposure to treatment, about one-third of patients with organ-confined PCa fail surgical treatment and progress to aggressive or metastatic lethal disease in 10 years [2]. Therefore, understanding mechanisms underlying the tumorigenesis and development of PCa is needed for early diagnosis, risk assessment and selection of therapeutic strategies against PCa. In this study, we identified KIF14 as a potential candidate oncogene involved in tumor progression and poor prognosis in prostate cancer.

KIF14 is a member of kinesin family proteins involved in cell division, microtubule polymer dynamics, intracellular transportation and signal transduction [3]. There is increasing evidence that KIF14 is overexpressed in multiple types of tumors, including hepatocellular carcinoma, lung cancer, breast cancer, etc [6–12], implicating its role as an potential oncogene. The upregulation of KIF14 might be caused by genomic amplification [6, 9, 12]. It was reported that in hepatocellular carcinoma, loss of KIF14 downregulates the expression of Skp2 and Cks1, leading to accumulation of p27Kip1. Downregulation of Skp2 and Cks1 also resulted in cytokinesis failure, which may inhibit tumor growth [6]. Inhibition of KIF14 inhibited the migration and induces apoptosis via inactivation of Akt kinase in HCC cells [7]. The effect of KIF14 on AKT phosphorylation was also reported in triple-negative breast cancer [9]. However, the expression of *KIF14* and its role in prostate carcinoma remain unclear thus far. The present study demonstrated for the first time, to our knowledge that *KIF14* is a candidate oncogene in PCa, which is upregulated in multiple cell lines of PCa and primary human PCa tissues. Remarkly, *KIF14* depletion induced cell cycle arrest, reduced proliferation and apoptosis in PCa cells at least partially through upregulating *GADD45A*, *GADD45B*, *p21, PIDD* and *Shisa5*, which contribute to growth arrest and apoptosis induction, and downregulating *CCNB1* that promotes cell cycle progression.

GADD45 mediated G2/M checkpoint in human and murine cells [28], this effect may be due to GADD45 ability to dissociate complexes of Cyclin B1 and Cdc2 [29]. The dephosphorylation of the CDC2/CCNB complex mediated by Cdc25 allows cells entry into M phase [30, 31]. The main function of p21 in cell cycle regulation is inhibiting the activity of cyclin B/Cdk1 complexes. Thus p21 is responsible for the nuclear retention of the cyclin B/Cdk1 complex and cyclin B degradation in response to DNA damage [32]. In our study, we found CCNB was repressed, while GADD45A, GADD45B and p21 were upregulated after KIF14 inhibition. The changed expression profile of these genes could explain G2 arrest in *KIF14* knockdown cells. G2 arrest will in turn lead to lower proliferation rate in cells and it could explain the inhibition of proliferation in KIF14 knockdown cells. We also found that SKP2 was downregulated in our microarray study (data not shown), which is consistent with other group's study [6]. We also found that PIDD, which interact with other death domain proteins and function as an adaptor protein in cell death-related signaling processes [33], was upregulated in KIF14 downregulated cells. PIDD is an effector of p53-induced apoptosis and it could act as switch between cell survival and cell death in response to DNA damage [33]. Shisa5 is another apoptotic protein upregulated in KIF14 knockdown cells. Shisa5 is localized to the endoplasmic reticulum and together with p53 induces apoptosis in a caspase-dependent manner [34, 35]. The upregulation of these two proteins will induce apoptosis in KIF14 knockdown cells. Our future study will be focused on how KIF14 regulates those genes’ expression.

Except for KIF14, many other kinesin proteins are reported as oncoproteins [36–43]. For example, KIFC1 induces resistance to docetaxel and is associated with survival of patients with prostate cancer [36]; KIF11 is a driver of invasion, proliferation, and self-renewal in glioblastoma [37]; KIF3C promotes tumor growth and metastasis in breast cancer by regulating TGF-β signaling [38]; It is also reported that inhibition of KIF22 suppresses cancer cell proliferation by delaying mitotic exit through upregulating CDC25C expression [39]; KIF3a can promote proliferation and invasion via Wnt signaling in advanced prostate cancer [40]. All these further support our finding that KIF14 is a functional oncoprotein in prostate cancer.

It has been reported that KIF14 is a marker of poor prognosis in patients with hepatocellular carcinoma and ovarian cancer, where *KIF14* overexpression enhances tumor growth, while its knockdown decreases tumorigenicity *in vitro* and in xenografts [13]. However, the role of KIF14 as an indicator in PCa is largely unknown. Here, we found that KIF14 was upregulated in most clinical PCa tissues and cell lines. Subsequently, by immunohistochemical methods, we observed a positive correlation between KIF4 expression and histological grade, clinical stage and Gleason Score. Kaplan-Meier analysis demonstrated that KIF14 was a prognostic factor for patient survival. Our results thus indicate that KIF14 could be served as a novel and useful prognostic biomarker for PCa.

In summary, our study found that KIF14 is upregulated in PCa cell lines and clinical PCa tissues. Inhibition of *KIF14* suppresses cell proliferation, induces G2 arrest and apoptosis. This effect was mainly caused by regulating GADD45A, GADD45B, CCNB, p21, PIDD and SHISA5 expression. Moreover, the protein level of KIF14 is positively correlated with histological grade, clinical tumor stage and poor prognosis in patients with PCa. Thus, our results suggest that KIF14 could act as a candidate oncogene that contributes to tumor progression and poor prognosis in PCa. KIF14 may represent a novel molecular target for the treatment of PCa.

## MATERIALS AND METHODS

### Cell culture and transfection

, DU145 and PC3) were used for this study. All cells were purchased from ATCC. Cells were maintained in RPMI 1640 medium (Invitrogen, Carlsbad, CA) containing 10% fetal bovine serum (FBS), 1% penicillin–streptomycin (10 ng/ml penicillin and 10 U/ml streptomycin), and 2.5 mM glutamine at 37°C in a humidified 5% CO2 incubator [44]. KIF14-short interfering RNA (siKIF14, Santa Cruz biotechnology, catalog no: sc-60882) and control siRNA (siControl, Santa Cruz biotechnology, catalog no: sc-37007) were purchased from Santa Cruz Biotechnology (Dallas, TX, USA). Transfection was carried out according to the manufacturer's instruction using RNAiMAX transfection reagent (Invitrogen, Eugene, OR, USA, catalog no: 13778).

### RNA extraction, reverse transcription and quantitative realtime PCR

RNA was extracted with Trizol reagent according to manufacturer's protocol (Invitrogen, Eugene, OR, USA). One normal prostate RNA was purchased from Clontech (Clontech, Palo Alto, CA). Reverse transcription was performed with Random hexamers and SuperScript-III (Invitrogen, Eugene, OR, USA). Quantitative real-time PCR was carried out with the Applied Biosystems 7300 real-time systems using real-time PCR Master Mix (SYBR Green). The standard curve was used to establish amplification efficiency [45]. All primers used are listed in Table 3. Each experiment was performed in triplicate in three independent experiments.

**Table 3:** Primer list used in this study

Application	Primer name	Sequence (5′→3′)	Size (bp)
Realtime PCR	KIF14F	TTCAGAACACCTCTGCAGGA	128 bp
	KIF14R	ACTCATGAAGACTACCTGGG	
	GAPDHF	TCATTGACCTCAACTACATG	131 bp
	GAPDHR	TCGCTCCTGGAAGATGGTGAT	
	P21F	AGACCAGCATGACAGATTTC	140 bp
	P21R	ACTGAGACTAAGGCAGAAGA	
	CCNBF	CCTCCGGTGTTCTGCTTCTC	122 bp
	CCNBR	GCCTGCCATGTTGATCTTCG	
	GADD45AF	CTTGGAGACCGACGCTGG	149 bp
	GADD45AR	TGTAGCGACTTTCCCGGC	
	GADD45BF	ATCAACATCGTGCGGGTGT	122 bp
	GADD45BR	GTGTGAGGGTTCGTGACCAG	
	PIDD1F	TGTTCGAGGGCGAAGAGTTC	148 bp
	PIDD1R	TCCAGAGTGGTGGTCACGTA	
	SHISAF	GAAAGGTGTGCTGTGCCTGA	105 bp
	SHISAR	TGACATGGGGTCGTTGTAGC	

### Growth curve

DU145 or PC3 cells were seeded in six-well plates at an appropriate density with penicillin/streptomycin free medium. Cells were transfected with control siRNA or siKIF14, respectively. After transfection, cell numbers were counted every 24 hours including the start point. Each experiment was conducted in triplicate in three independent experiments.

### Flow cytometry analysis of cell cycle

About 48 hours after transfection, cells were harvested and fixed with ice-cold 70% ethanol for 24 hours. After that, cells were washed with PBS once and stained with 100 μl of 50 mg/L propidium iodide (PI) for 15 min at 4°C in dark. The cell cycle profiles were assayed by flow cytometry (excitation at 536 nm and emission at 617 nm) with a flow cytometer (FACS Calibur^TM^, BD Biosciences, CA, USA) and data were analyzed with the ModFit LT software. Each experiment was conducted in triplicate in three independent experiments.

### Apoptosis assay

Apoptosis was assessed by flow cytometry analysis. Apoptotic cells were detected with PE Annexin V Apoptosis Detection Kit I (BD Bioscience). About 48 hours after transfection, cells were harvested and centrifuged at 1000 rpm for 10 min, rinsed with cold PBS once. About 1 × 10^5^ cells were stained with 5 μl PE Annexin V and 5 μl 7-AAD working solution for 15 min at room temperature in dark. The ratio of apoptotic cells were analyzed by flow cytometry. The total number of early apoptotic cells and late apoptotic cells were considered as total apoptotic cells. Each experiment was conducted in triplicate in three independent experiments.

### RNA sequencing

RNA was extracted with an RNeasy Mini kit (QIAGEN). Firstly, mRNA was purified and RNA-Sequencing library preparation was performed according to the manual of manufacturers (KAPA biosystems). Sequencing reactions were performed with the Illumina HiSeq platform. RNA-seq reads were mapped to the human genome (hg19) using Burrows-Wheeler Aligner (bwa). The duplicate reads were then marked by picard. The HTseq tool was used to calculate the reads count for each gene. Finally, we used the Reads Per Kilobase per Million mapped reads (rpkm) command in edgeR package to calculate the rpkm of each gene. Specifically, we added one to the reads count for each gene and take log values. These log values were inputted into the heatmap.2 in R package gplots to generate the heatmap figure [46]. The genes with 2 fold changes were loaded to David bioinformatics database (https://david.ncifcrf.gov/) for pathway analysis.

### Oncomine analysis

The Oncomine cancer microarray database (http://www.oncomine.com) [14] was used to analyze expression profiles of KIF14 in a variety of human prostate cancer and normal tissues. Two representative prostate cancer datasets [15, 16] were presented in this study. A fold-change of 2 for KIF14 gene expression compared to control was required for criterion.

### Patient samples and IHC

All patient samples were collected in Shenzhen People's Hospital with the permission of patients. All PCa and benign prostatic hyperplasia (BPH) specimens were obtained immediately after operation (radical prostatectomy, transrectal biopsy or transurethral resection of the prostate). Tissues were then fixed in 10 % buffered formalin and embedded in paraffin. Tissue sections were stained with KLF14 antibody purchased from abcam. The protocol of IHC staining was described before [47]. The clinical characteristics of the PCa cohort are listed in Table 2. The tumor staging and differentiation of PCa was defined according to the 2010 AJCC Cancer Staging Manual seventh edition. (Well differentiation: Gleason Score ≤ 6) Moderate differentiation: Gleason Score = 7; Poor differentiation: Gleason Score ≥ 8)

### Follow-up

All patients with PCa received check-ups every 2–3 months during the first 2 years and every 3–6 months there after until the follow-up period ended in January 2017. Surgeons who were blinded to the study performed the follow-up study. The overall survival was defined as the length of time between surgery and either the death of the patient or the last follow-up visit.

### Statistical analysis

Data are presented as mean ± standard deviation. Statistical assessments were carried out using Student's *t* test. *P* < 0.05 was considered statistically significant.
